# Liability of a Lunatic's Estate for Necessaries Supplied to the Lunatic

**Published:** 1851-04-01

**Authors:** 


### LIABILITY OF A LUNATIC'S ESTATE FOR NECESSARIES SUPPLIED TO THE LUNATIC.

### Seaton *v.* Adcock.

This was an action tried in the Court of Queen's Bench, before Lord Campbell and a common jury, on the 13th of February last, and we are induced to notice it on account of some points in the case likely to be interesting to the proprietors of asylums and to others having to do with lunatics.

The action was brought by Dr. Seaton, the proprietor of a private asylum, against the administrator of the estate of a deceased gentleman who had been found lunatic by inquisition, and who had been a patient under Dr. Seaton's care (and who, it appears, died in Dr. Seaton's house about five years ago), for a balance due for the maintenance of the lunatic, and for other necessaries supplied to him, and also for sundry expenses incidental to the suing out of the commission, and which had been incurred under the authority of this very person who was now acting as administrator, but who was at that time acting as solicitor to the next of kin to the lunatic.

The patient, it appeared, had been duly placed under Dr. Seaton's care, upon the usual medical certificates, and an order signed by the lunatic's next of kin, his only sister, and who, it seems, paid the first quarter's maintenance which accrued previously to the holding the commission, the balance for which the action was brought having accrued subsequently to the inquisition.

The substantial defence set up was, that forasmuch as the contract was not entered into by the lunatic, his estate was not liable; and further, it was attempted to be shown that the contract for maintenance was specially made with Dr. Seaton by the lunatic's sister *on her own responsibility*, and the counsel for the defence also contended that even for necessaries the lunatic's estate would not be liable if they should have been supplied with the full knowledge that he was lunatic. The learned judge interrupting the counsel, inquired if there were any precedents for such a doctrine? at the same time observing, "I shall be very sorry to hear that there are;" adding, "I shall feel it my duty to direct the jury that such is not the law."

The propriety and moderation of the several items of the account were fully proved, —indeed, their justice and moderation were so palpable, that the learned judge more than once interfered and characterized the defence to the action, as being "little creditable to those who put it forward;" indeed, the absence of a *bonâ fide* defence was virtually acknowledged by the defendant in the attempt which was made by his counsel to palliate the obvious vexatiousness and injustice of the defence, by freely imputing unworthy conduct to the plaintiff—imputations which were subsequently proved by their own witness to be as unfounded as they were gross.

During the deceased's residence in the asylum, he executed a paper which he intended as a will, and therein he mentioned Dr. Seaton's name beneficially—devising him a house valued at 600*l*., but, with that exception, leaving the whole of his property to his next of kin, subject only to the payment of two small annuities (to old servants, we believe), and as executors of this will, or intended will, he nominated as one a gentleman in the Bank of England, who had already been executor to his deceased father and brother; and as the other, Dr. Seaton.

Out of these materials it was sought to find a palliation for the defence offered in the present action, by attempting to raise the assumption that the deceased must have been unduly influenced by Dr. Seaton; but no evidence was adduced showing a shadow of pretence for such an assumption, the defendant evidently trusting to the chances of his counsel being able by a damaging speech to divert the jury from the point they really had to try; how far such a course might have succeeded with a less intelligent jury, or with a judge less acute than Lord Campbell, we cannot tell; in the present instance, however, the effect was to draw down upon the defence the severest animadversion of the learned judge, and the assumption was most conclusively disproved by a witness for the defence, who had been put into the witness-box for another purpose.

To prove the plea that the estate was not liable because the lunatic did not enter into the contract for his maintenance, and also that there was a special contract with another person, the defendant put into the witness-box the sister of the deceased, upon whose authority Dr. Seaton had originally received the patient, and this witness swore that she had specially entered into the contract with Dr. Seaton *on her own responsibility*; in cross-examination, she admitted that she was a *married woman* separated from her husband, *who was living out of the country*. As a married woman, of course she could not be sued; consequently, had the jury believed her evidence, and found accordingly, the plaintiff would have had no remedy, and, worse still, all the costs of this action must have fallen upon him; and, what made the bare-facedness of this defence the more iniquitous was the fact, that this very witness was the *real* present defendant,—an officer from the Prerogative Court produced the letters of administration, and proved that the defendant had administered to the estate as solicitor to Elizabeth Germani (the witness) and "for and on her behalf." Being a married woman, and her husband out of the country, we presume she could not herself administer.

In answer to questions from the defendant's counsel, in reference to the will he had alluded to in his speech, this witness stated, that it had cost her a considerable sum of money to set the will aside, and broadly stated that it was Dr. Seaton who had put her to this expense. In cross-examination, she admitted that immediately upon her brother's death, Dr. Seaton had gone to London, produced the paper to her, and told her that of course he should not take any step (as one of the executors named therein) to prove the will, and intimated that if it were proved he should not wish to take any benefit under it, and declared his willingness forthwith to execute a "release" of all claim under it.

She admitted that, in accordance with this declaration, Dr. Seaton subsequently did actually decline to propound the will, and that he and the other executor having "renounced," the will was ultimately propounded by one of the legatees.

Hereupon the learned judge asked the witness, "What then did Dr. Seaton do to prove the will?" After some little hesitation the witness replied, "I don't know."

Lord Campbell then addressed the jury as follows; "Gentlemen, in this case a most unfair advantage has been taken by the defendant of the circumstance of the lunatic having made a will while at the plaintiff's house. It is most discreditable to defend such an action on such grounds. If Dr. Seaton had sought to take advantage of a will made under such circumstances, his conduct would have been infamous; but it is proved by the defendant's own witness that Dr. Seaton did all that an honourable man could have been expected to do; immediately after the death of the lunatic he went to the sister, and offered to 'release' all interest under it. What more could he have been expected to do? You have been told that the estate of a lunatic is not liable for even necessaries, if the person supplying them is aware of the lunacy at the time; it is my duty, however, to tell you that such is not the law." His lordship then proceeded to point out some of the serious evils which must happen if such a doctrine were to prevail, and continued: "There can be no doubt that the estate of a lunatic is liable for necessaries, although supplied with a full knowledge of the lunacy; and by necessaries must be understood things fitted to the station of the individual; if, however, there has been a special contract with some other person, the estate would be exonerated from liability; therefore, in this case, if you are of opinion that the last witness did, as she states, make this contract with Dr. Seaton upon her own responsibility, you will, of course, find a verdict for the defendant generally; but if you are of opinion—as I think it probable you will be—that this lady really acted in the character of *agent for her brother*, he being necessarily incompetent to act for himself, it will be your duty to find a verdict for the plaintiff for such sum as you may consider him entitled to."

His lordship then referred to the items in the account, observing that they appeared to be perfectly fair and reasonable, and such as ought to be paid; but that as regarded some of them, he did not think in law the estate could be held liable.

He then proceeded to go through them seriatim. The first item for which the learned judge ruled that the estate could not be held liable, was a charge of seven guineas for attending in London to make an affidavit, in compliance with a letter (read in court) from the present defendant, who was then acting as solicitor for the sister, suing out the commission; this charge of seven guineas was inclusive of travelling expenses from Coventry to London and back. The judge held, that as the commission was a proceeding adverse to the lunatic, his estate was not liable for expenses incurred in it.*

The next item was also a charge of seven guineas, incurred a few days afterwards for the same purpose, and under exactly the same circumstances; of course, it was disposed of in like manner.

The next item was a sum of twenty-two guineas, for bringing the lunatic from Coventry to London, to be present at the inquisition, and remaining with him in town two days, and for being examined as a witness at the inquisition. This item, also, the learned judge, with evident reluctance, told the jury that the estate could not be held liable for.

Then came two sums of five guineas, which Dr. Seaton had paid to a London physician for visiting the deceased on two occasions when he was urgently ill.† The

---

* Although by no means pretending to be "learned in the law," we cannot help thinking that the point here involved is open to discussion. If a commission fails, the presumption is that the individual is *not* of unsound mind, and in that case an attempt to prove him so would undoubtedly be an adverse proceeding; but where the man *is really* a lunatic, we apprehend that a commission *de lunatico inquirendo* can hardly be taken to be an adverse proceeding, seeing that is the only means by which protection for his person and property can be adequately secured. To protect the person and interests of a man who is himself unable to protect them, must surely be doing him a service; and a witness who testifies to his lunacy should, we think, be considered as a witness *on behalf of* instead of against the lunatic, and, as such, would have an equitable claim, and should have a legal claim upon the estate for his services; indirectly, such claim against the estate is admitted in the present instance. Had the next of kin who sued out the commission paid the plaintiff these charges at the time (as is usual), they would have been repaid to her out of the estate, under the usual order of the Lord Chancellor, in such cases. It is no doubt *customary* for the person suing out a commission, in the first instance to pay the costs and charges of the various persons who may have been employed in the matter, and then for the Chancellor, upon the report of the Master, to order the repayment out of the estate, and doubtless that is the most *convenient*, and for the most part the necessary arrangement; but we can see no reason why the Chancellor could not, if he were so disposed, or if it were necessary, order payment of those costs and charges *directly* to the several parties, for surely if (having been paid) it be legal to charge the estate with their *re*-payment, it must be equally legal (not having been paid) to charge the estate with their payment. The real questions appear to us to be, was the person (witness or other) *properly* employed in the matter? and is the charge *moderate* and proper?—Ed.

† Dr. Seaton had then removed from Warwickshire to Sunbury.

physician to whom it was paid proved that he had received the ten guineas three years ago; that, however, was not until *after the patient's death*, on which account the learned judge was of opinion that the estate was not liable; but in giving that opinion to the jury Lord Campbell observed, "I am sorry to be obliged to tell you so, as I think it very hard upon Dr. Seaton, but this is one of those cases where, unfortunately, law is not justice."

After going through the remaining items, consisting of the balance due for maintenance at the rate of 200*l.* per annum, and some trifling sums involving no point of importance, the learned judge left the case to the jury, who immediately returned a verdict for the plaintiff for 139*l.* (the sum claimed we understood to be 145*l.*) The jury were then asked how they made up the amount, when it was found that they had set a higher value upon justice than law, by including in their verdict most of the sums which the learned judge had ruled could not in law be recovered against the estate. This must have been very satisfactory to the plaintiff, as showing the opinion the jury entertained of the discreditable character of the defence; but as the probable result would have been further litigation (the defendant might next term have moved for a new trial), under the advice of the learned judge a reduced verdict was taken "by consent."

Mr. Serjeant Shee, Mr. Wordsworth, and Mr. Prentice (instructed by Messrs. May and Sweetland, solicitors) were counsel for the plaintiff.

Mr. Knowles and another gentleman, for defendant.

